# Cholera

**Published:** 1849-01

**Authors:** 


### CHOLERA.

We are happy to say, that the alarm which existed some two weeks since, has, in a great measure, subsided. It is really astonishing how many extravagant reports get into circulation. We hear almost daily from different sections of the country, and the news which thus flows in upon us, gives us the number of deaths daily in this city, at from 50 to 150.

That the true state of the case may be known, we give below the report of the Board of Health of the 10th inst. It will also be seen, that the city authorities are preparing to meet the calamity when it may come—which we hope may be long postponed.

That which appears to strip the disease of its most alarming feature, is—cleanliness, proper diet, regular rest, and due attention to premonitory symptoms; but, above all, let not a continued brooding over apprehensions of this disease, bring on these symptoms.—[Cin. Ed.

City Council. Wednesday Evening, Jan. 10th.

No business of particular importance was transacted. An ordinance respecting the cleaning of the streets by contract, was reported, and made the special order of the evening for Friday. The Board of Health reported that Dr. Muscroft has been appointed Actuary or Health Officer, by the Board; that a Cholera Hospital has been established at the Bethel Chapel, on East Front street, and requesting authority to establish others. The Committee reported that about twenty deaths from Cholera, in all, have been reported; one-third of which were cases which originated here, and the others imported; and that six cases are reported to be now existing; but the Board believe the favorable state of the weather will check the existence of the disease. The report was referred to the Committee on Health.—*Atlas.*

Jan. 17th.—Since the above report from the Board of Health, no new cases have occurred; and from the best information we now have, we can say that our city is free of this disease. This, with the fact that the disease has nearly left New Orleans, and is, so far as we know, subsiding between this and that city, leads us to believe that we shall not have it as an epidemic before next spring or summer, if then.—[Cin. Ed.

